# TALEN-mediated *apc* mutation in *Xenopus tropicalis* phenocopies familial adenomatous polyposis

**DOI:** 10.18632/oncoscience.166

**Published:** 2015-05-19

**Authors:** Tom Van Nieuwenhuysen, Thomas Naert, Hong Thi Tran, Griet Van Imschoot, Sarah Geurs, Ellen Sanders, David Creytens, Frans Van Roy, Kris Vleminckx

**Affiliations:** ^1^ Developmental Biology Unit, Department of Biomedical Molecular Biology, Ghent University, Ghent, Belgium; ^2^ Molecular Cell Biology Unit, Department of Biomedical Molecular Biology, Ghent University, Ghent, Belgium; ^3^ Inflammation Research Center, Flanders Institute for Biotechnology (VIB), Ghent, Belgium; ^4^ Department of Pathology, Ghent University and Ghent University Hospital, Ghent, Belgium; ^5^ Center for Medical Genetics, Ghent University and Ghent University Hospital, Ghent, Belgium

**Keywords:** APC, Intestinal cancer, desmoid tumors, Wnt signaling, animal model

## Abstract

Truncating mutations in the tumor suppressor gene adenomatous polyposis coli (APC) are the initiating step in the vast majority of sporadic colorectal cancers, and they underlie familial adenomatous polyposis (FAP) syndromes. Modeling of APC- driven tumor formation in the mouse has contributed substantially to our mechanistic understanding of the associated disease, but additional models are needed to explore therapeutic opportunities and overcome current limitations of mouse models. We report on a novel and penetrant genetic cancer model in *Xenopus tropicalis*, an aquatic tetrapod vertebrate with external development, diploid genome and short life cycle. Tadpoles and froglets derived from embryos injected with TAL effector nucleases targeting the *apc* gene rapidly developed intestinal hyperplasia and other neoplasms observed in FAP patients, including desmoid tumors and medulloblastomas. Bi-allelic *apc* mutations causing frame shifts were detected in the tumors, which displayed activation of the Wnt/β-catenin pathway and showed increased cellular proliferation. We further demonstrate that simultaneous double bi-allelic mutation of *apc* and a non-relevant gene is possible in the neoplasias, opening the door for identification and characterization of effector or modifier genes in tumors expressing truncated apc. Our results demonstrate the power of modeling human cancer in *Xenopus tropicalis* using mosaic TALEN-mediated bi-allelic gene disruption.

## INTRODUCTION

FAP is an autosomal dominant disorder characterized by the presence of hundreds to thousands of benign, adenomatous polyps in the colon, which over time can progress to malignant adenocarcinomas. FAP is caused by germline mutations in the *APC* gene. Different variants of FAP exist. In the Gardner syndrome (OMIM 175100, 135290), gastrointestinal adenoma formation is frequently accompanied by extra-colonic manifestations, such as congenital hypertrophy of the retinal pigment epithelium (CHRPE), desmoid tumors, osteomas, dental anomalies, epidermal cysts, and soft tissue tumors. In patients diagnosed with the Turcot syndrome (OMIM 276300), central nervous system malignancies, such as medulloblastomas, are also observed. Although prophylactic colorectal surgery significantly reduces the mortality associated with FAP, the other less penetrant signs and symptoms are becoming more clinically relevant, most prominently the desmoid tumors, which are hard to treat and are a major cause of death in FAP patients [1, 2].

Nonsense mutations in the *APC* gene underlie FAP, and these mutations are also observed in most sporadic colon cancers. Several mouse FAP models have been identified or generated [3], the most often used model being the Apc^Min^ mouse. Conditional models with floxed *Apc* alleles have also been generated [3]. Recently intestinal tumor formation has also been described in rats and zebrafish carrying ENU-induced mutant *Apc* alleles [4, 5].

Mutations in the *APC* gene result in the expression of a truncated APC protein that causes ectopic constitutive activation of the Wnt/β-catenin pathway, which is seen as an important target for therapeutic intervention. Unfortunately, the Wnt pathway is notoriously difficult to target by small chemical compounds [6, 7]. Many screening efforts have so far not yielded any applicable drug urging the need for alternative animal models such as *Xenopus* that allow fast and reliable screening of novel therapeutics or potential therapeutic targets [8, 9].

Modeling human disease in non-mammalian vertebrates has been primarily restricted to zebrafish, which has been subjected to large-scale mutagenesis screens [10, 11]. Therefore, zebrafish is being increasingly used as a cancer model [12]. Until now, *Xenopus* has not reached the same status because it has been used mostly for transient approaches such as RNA and Morpholino injections. However, TALEN and CRISPR/Cas9 mediated genome editing is creating a revolution in the field of functional genomics [13], opening the door for many novel experimental approaches and strategies in numerous model organisms. It is easy and economical to make custom TALEN and CRISPR/Cas9 constructs, and the technologies are within reach of any laboratory with standard molecular biology facilities. Moreover, zebrafish and *Xenopus* are very suitable for knockout experiments because they develop externally and require simple injection setups, thus allowing cheap semi-high- throughput gene knock-out approaches. Importantly, unlike *X. laevis* and zebrafish, *X. tropicalis* has a true diploid genome and can therefore gain in importance for modeling human diseases, including cancer [14].

Here, we describe the first genetic cancer model in *X. tropicalis*. Using TALEN-mediated targeting of the mutation cluster region (MCR) of the *apc* allele, we developed a model that mimics the human FAP syndrome and remarkably recapitulates several of the tumor types observed in FAP patients. This model may pave the way for establishing other cancer models in *X. tropicalis* and for its use in pre-clinical drug screening.

## RESULTS

### Design and use of a TALEN construct targeting the apc MCR in *Xenopus tropicalis*

**Figure 1:**
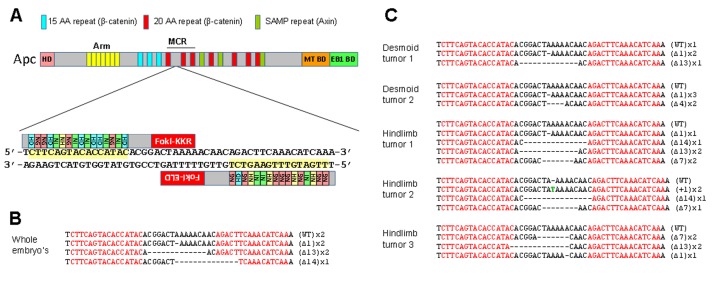
TALEN mediated targeting of the MCR of the Xenopus tropicalis *apc* gene (A) Schematic representation of the apc protein, the region that was targeted by TALENs, and the TALENs used. (B) Deletions detected in the targeted region of *apc* gene in embryos injected with 300 pg of *apc* TALEN mRNA. The sequences in red indicate the *apc* TALEN recognition sites. (C) Bi-allelic mutation of the *apc* gene identified in desmoid and hindlimb tumors dissected from tadpoles having undergone *apc* TALEN mRNA (40 pg) injection. AA, Amino Acid; APC, Adenomatous Polyposis Coli; Arm, Armadillo repeats; MCR, Mutation Cluster Region; EB1 BD, End Binding protein 1 Binding Domain; HD, Homodimerization domain; MT BD, Microtubule Binding Domain.

We first determined the domain structures of the *X. tropicalis apc* gene and its encoded protein. The structural and the functional domains of the frog APC protein are very similar to their human counterparts, including a potential N-terminal homodimerization domain, an armadillo repeat region, three repeats of 15 amino acids (aa), seven 20-aa repeats responsible for binding β-catenin, three SAMP repeats known to interact with axin, a microtubule-binding domain, and a C-terminal EB-1 binding region (Fig. 1A) [15]. Importantly, as in mammals, the last exon of the *apc* gene is very long, encoding three-fourths of the protein. Therefore, nonsense or frame-shift mutations in the sequence downstream of the armadillo repeats escape nonsense-mediated decay, and so can generate truncated proteins.

In humans, most truncating *APC* mutations occur between the first and third 20-aa repeats [15]. We first sequenced this region from four frogs in our colony to detect any single nucleotide polymorphisms (SNPs) that might interfere with TALEN binding, but none were found. We then designed a TALEN construct to target a sequence between the first and the second 20-aa repeats. An *apc* TALEN pair was assembled using Golden Gate cloning [16]. Each TALEN arm was fused to either ELD or KKR mutant Fok1, which is active as an obligate heterodimer, hence increasing specificity [17]. No off-target sites were predicted by the TALEN-targeter software [18].

Synthetic RNAs generated from the *apc* TALEN pair were micro-injected into two-cell stage embryos, which were then grown until stage 46. Individual embryos were lysed and genomic DNA was extracted. The targeted region was amplified and subcloned into pTOPO plasmids. Sequencing of individual plasmid clones revealed discrete insertion and deletion mutations (INDELs) in 59% (n= 17) of the clones (Fig. 1B). This demonstrates that the *apc* TALEN strategy is very efficient in *X. tropicalis* allowing introduction of frame-shifting INDELs in the *apc* MCR region. These mutations are predicted to result in expression of truncated proteins, which mimic the human disease-prone situation.

### Formation of tumors with bi-allelic apc mutation

High doses (300 pg) of *apc* TALEN were largely detrimental to embryonic development, most likely because they disrupt several early developmental processes that are under control of Wnt/β-catenin signaling. However, a large portion of embryos injected with lower doses (40 pg) progressed into the tadpole stage. When the tadpoles developed their hind limbs, several external neoplastic lesions developed. Most striking were the many cyst-like tumors in the epidermis, several subcutaneous tumors, and large, fast-growing tumors in the region of the extending limbs.

APC-driven intestinal tumor formation in humans is associated with bi-allelic mutations or loss of the wild type copy evidenced by loss of heterozygosity [19]. So we analyzed whether the tumors appearing in the tadpoles injected as an embryo with *apc* TALENS had two mutant *apc* alleles. Indeed, in each of the five subcutaneous and hind limb tumors that we analyzed, at least two different INDEL mutations in the *apc* MCR were apparent (Fig. 1C). All INDELs observed induced frame-shifts (*e.g.* +1, ß4, ß14). This is in line with the expectation that a subpopulation of the embryonic cells will develop truncating bi-allelic mutations that enable them to hyper- proliferate and form tumors.

**Figure 2:**
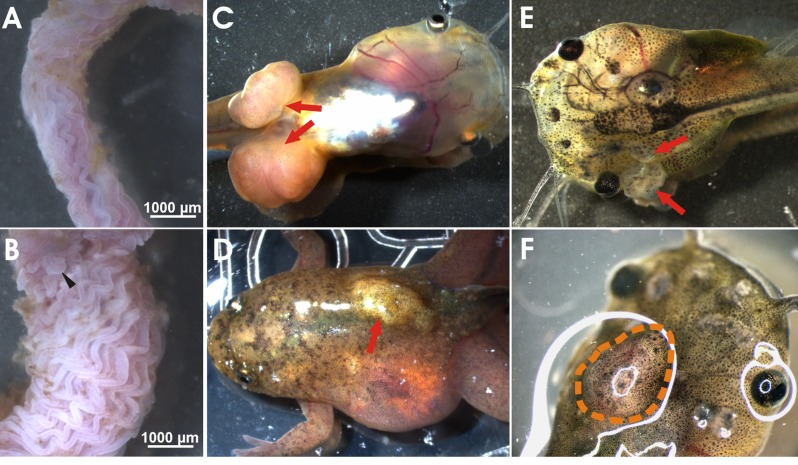
Neoplastic phenotypes observed in *apc* TALEN mRNA injected tadpoles and frogs Small intestine, cut open longitudinally, of wild type (WT) (A) and *apc* TALEN mRNA (40 pg) injected (B) adult frogs. In WT frogs, the epithelial lining of the duodenum is organized in parallel longitudinal folds. The intestines of *apc* TALEN injected animals are largely expanded and the intestinal folds are irregular and excessively undulated. Aberrant local protuberances are visible (black arrowhead). (C) Large fast growing tumors at the position where the hindlimbs emerge (red arrows). (D) Desmoid tumor, visible as a subcutaneous light colored mass (red arrow). (E) Epidermoid cysts (red arrows) originating at various positions in the tadpole skin. (F) Medulloblastoma visible as a large mass in the tadpole brain region.

**Table 1:** Overview of the externally visible tumor phenotypes in animals injected at the 8-cell stage with 20 pg of *apc* TALEN mRNA in a single blastomere

	Animal-Dorsal			Animal-Ventral			Vegetal-Dorsal			Vegetal-Ventral		
	5 weeks	9 weeks	18 weeks	5 weeks	9 weeks	18 weeks	5 weeks	8 weeks	18 weeks	5 weeks	8 weeks	18 weeks
	n=14	n=7	n=4	n=19	n=10	n=5	n=18	n=14	n=7	n=18	n=7	n=1
**Epidermoid cysts**	**21%**	**14%**	**0%**	**47%**	**30%**	**0%**	**0%**	**0%**	**0%**	**5%**	**14%**	**0%**
**Desmoid tumors**	**0%**	**0%**	**0%**	**0%**	**50%**	**0%**	**0%**	**71%**	**86%**	**0%**	**71%**	**100%**
**Limb tumors**	**0%**	**0%**	**0%**	**0%**	**0%**	**0%**	**0%**	**0%**	**0%**	**33%**	**57%**	**0%**
**Survival (beyond 5 weeks)**		**50%**	**29%**		**53%**	**26%**		**78%**	**39%**		**39%**	**6%**

### Intestinal and extra-intestinal hyperplasia and malformations in mosaic apc mutant tadpoles and froglets

The mosaic *apc* mutant tadpoles and froglets rapidly developed externally visible neoplastic lesions. Most recurrent were subcutaneous desmoid tumors (55%, n=26), epidermoid cysts, fast growing tumors in the limb region (32%) and visible abnormalities in the brain (68%) and the eyes. The animals were euthanized when they showed clear signs of discomfort. This was especially the case for animals with the limb tumors, which grew fast to a very large size. This prevented the tadpoles form reaching metamorphosis. Hence, to increase tadpole survival, we performed targeted injections of the *apc* TALEN RNAs in unique individual blastomeres at the 8-cell stage. *Xenopus* embryos have well characterized fate maps, which allows to enrich the injected substances, such as the TALEN RNAs, in specific tissues and organs [20]. Embryos were injected in a single dorsal-animal, ventral-animal, dorsal-vegetal or a ventral-vegetal blastomere and the resulting tadpoles were observed for over 18 weeks. Interestingly, different tumor types were clearly linked to specific injection sites (Table 1). Epidermoid cysts were especially observed in embryos targeted in a ventral-animal or a dorsal-animal blastomere, which contribute primarily to ectodermal derived tissues such as the epidermis. In contrast, desmoid tumors were only observed upon injection of a dorsal-vegetal or ventral-vegetal blastomere, which contribute to the endodermal and mesodermal derived tissues and organs. The fast growing limb tumors were restricted to embryos injected in a ventral- vegetal blastomere, which contributes to the lateral plate mesoderm, from which the limb buds originate. Therefore, subsequent exclusion of injections in the ventral-vegetal region can prevent the fast growing limb tumors and hence increase the number of animals that reach metamorphosis.

**Figure 3:**
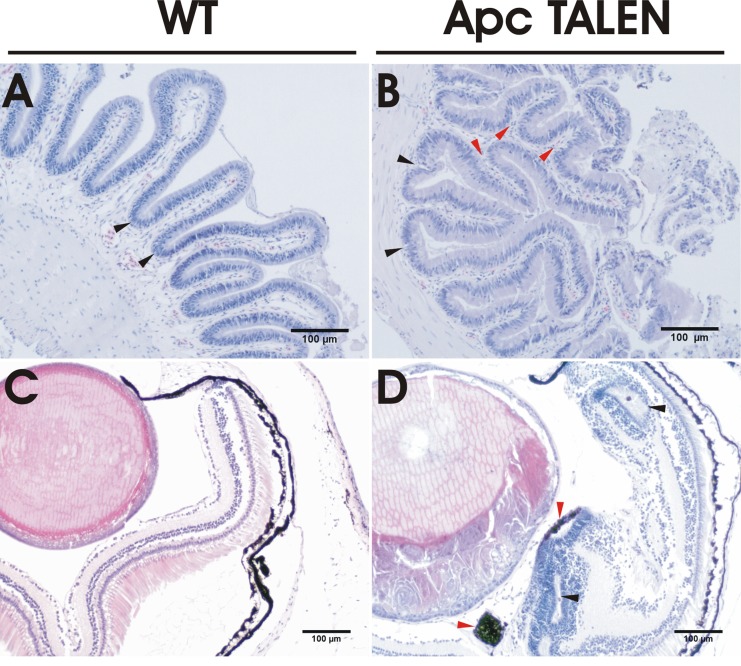
Histological analysis of intestinal and retinal sections (A) Haematoxylin and eosin (H&E) staining on a transversal section of the duodenum of a wild type frog. The mucosa is organized in regular alternating long and short folds and troughs (black arrowheads). (B) The small intestine of *apc* TALEN mRNA (40 pg) injected frogs shows irregular, branched folds (red arrowheads) and expanded troughs (black arrowheads). (C) Cross section through the eye of a WT tadpole showing the lens and the spatially organized retinal layers. (D) The eye of an *apc* TALEN mRNA (40 pg) injected tadpole displays hyperproliferation and abnormal layering of the neural retina showing rosette-like structures (black arrowheads) and ectopically localized retinal pigment epithelium (red arrowheads). In addition lens differentiation is affected.

**Figure 4:**
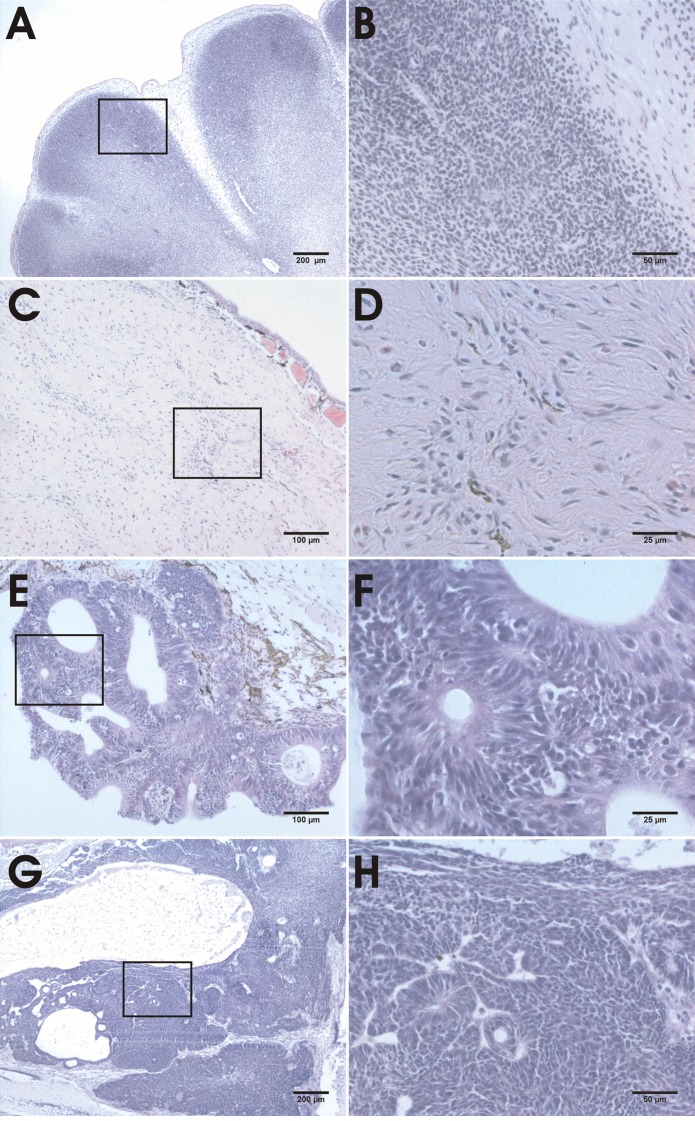
Histological analysis of hind limb, desmoid, epidermal and brain neoplasias of *apc* TALEN mRNA (40 pg) injected tadpoles and froglets The boxed regions in the left panels are shown at higher magnifications in the corresponding panels on the right. (A, B) Tumors associated with the developing hind limb are composed of uniform sheets of densely packed monotonously small, round cells with scant cytoplasm. (C, D) Desmoid tumor composed of spindle shaped or stellate fibroblastic cells interspersed with collagen deposits and with prominent vasculature. (E, F) Epidermoid cyst with neuroblastic elements, morphologically reminiscent of a malignant, poorly differentiated, primitive, embryonal neuroepithelial/neuroectodermal tumor (PNET). (G, H) Brain tumor with the histological characteristics of a medulloblastoma.

### Intestinal neoplasms

Intestinal organs were examined after opening of the peritoneal cavity. The liver was often enlarged and yellowish, but there was no evidence of tumor nodules (data not shown). The pancreas, kidneys and spleen did not show obvious macroscopic abnormalities. Although the small and large intestines in tadpoles did not show external signs of tumors, histological examination revealed abnormal organization of the intestine in tadpole stages (in two out of three intestines analyzed – data not shown). In adult frogs dissected at 10 months of age we observed local expansion of the intestine, especially in the duodenum (5 out of 15 intestines analyzed). This was especially apparent when the intestine was cut along the longitudinal axis and turned inside-out. The post-metamorphic intestinal cell layers are organized in longitudinal folds, and the trough-crest axis resembles the crypt−villus organization of mammalian small intestine. Lgr5-positive stem cells are present at the bottom of the troughs, again resembling the situation in mammals [21]. In contrast to wild-type frogs, the folds in the duodenum of animals injected with *apc* TALENs were massively expanded, irregularly structured and more undulated with local protuberances (Fig. 2A,B).

Histological examination of the intestine of *apc* TALEN injected post-metamorphic frogs revealed several abnormalities. Cross-sections of the normal intestine showed a thick muscularis externa lined by a mucosa evaginating into the lumen and forming multiple folds (Fig. 3A). These folds were tall and regular, with alternating long and shorter projections. The folds were lined by a monolayer of tall columnar cells overlying a lamina propria, which was in direct contact with the muscularis externa. The epithelial cells at the base and between the folds had a larger, darker stained elongated nucleus in a basal location. Towards the tip of the folds the nuclei were smaller and mucus-secreting goblet cells were present between the enterocytes.

Transversal sections of the intestine from the mutated animals showed expanded crypts and folds that were very irregular and of various length (Fig. 3B). Several folds showed ectopic crypts perpendicular to their longitudinal axis. The epithelial cells lining the cavity were small, but tall columnar cells with an elongated basal nucleus were present from the top to bottom of the folds and the number of cells lining the folds was increased relative to the normal samples. Features of inflammation were not observed in either normal or mutated samples.

### Limb associated tumors

Rapidly growing tumors with external lobular morphology appeared in the hind limb region of 30% of tadpoles injected at the 2-cell stage, often displacing the developing limb (Fig. 2C). A higher incidence (> 70%) of limb tumors (including tumors in the front limb region) was observed upon targeted injection of a ventral-vegetal blastomere (Table 1). These tumors were sometimes very large, with the inward growing part displacing the organs in the abdomen. Histological examination showed tumors with a solid and relatively circumscribed growth arrangement composed of patternless sheets of densely packed, monotonously small, round, cells with scant cytoplasm (SRBCT or small round blue cell tumors) (Fig. 4A,B). The most cell-dense areas were at the periphery. The central regions were more scarcely populated and contacted the forming limb structures. There was no discontinuity between the limb tissue and the neoplasm. This indicates that the tumor might have been derived from the limb mesenchyme.

### Desmoid tumors

A tumor type observed frequently in more than 30% of tadpoles injected at the 2-cell stage with *apc* TALENs was externally visible as subcutaneous light-colored masses (Fig. 2D). Tumor frequency surpassed 70% when the TALENs were injected at the 8-cell stage in a vegetal blastomere (Table 1). These tumors were relatively slow growing and became detectable in premetamorphic tadpoles (Fig. 2D). They were mostly attached to skin and/or muscles. Histology revealed a spindle-cell mesenchymal tumor composed of sweeping fascicles of uniform, spindle shaped or stellate, fibroblastic cells interspersed with collagen deposits and with a prominent vasculature (Fig. 4C,D). These features are characteristic of desmoid fibromatosis, a major extra-colonic tumor prevalent in FAP patients [22].

### Epidermoid cysts

The first tumors that were apparent in developing tadpoles were epidermal neoplasms, mostly detectable from two weeks of age on (Fig. 2E). The highest frequency (47%) was observed when targeting the TALENs to an animal-ventral blastomere, which contributes to the epidermis (Table 1). Histological examination revealed a cystic organization, also suggesting an epidermal origin (Fig. 4E). Although epidermoid cysts have been observed in FAP patients [22], their histological architecture is very different from the frog tumors. This may be due to differences in the organization of the skin, which in *Xenopus* tadpoles is not stratified. The cystic outgrowths contained neuroblastic elements with formation of rosettes, morphologically reminiscent of a malignant, poorly differentiated, primitive, embryonal neuroepithelial/neuroectodermal tumor (PNET). Areas of neuroepithelial differentiation were seen, with formation of primitive glandular structures resembling the neural tube and composed of pseudostratified, columnar-cuboidal epithelium (medulloepithelioma) (Fig. 4F).

**Figure 5:**
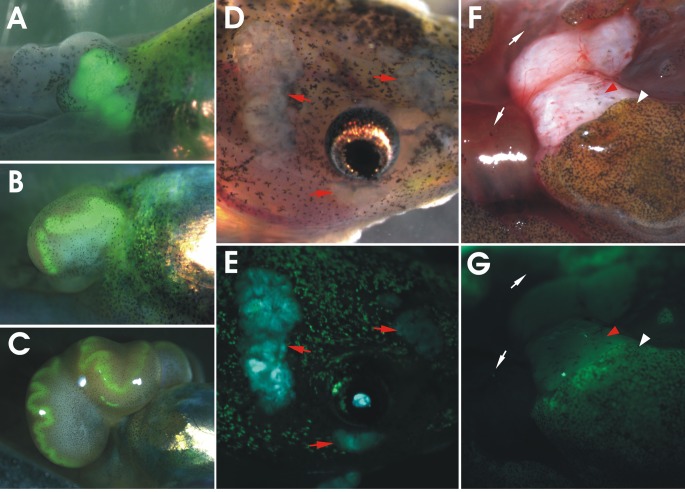
Active Wnt signalling in *apc* TALEN induced tumors GFP expression is increased in tumors developing in *apc* TALEN treated embryos derived from a transgenic Wnt reporter line [23]. (A-C) Intense GFP expression reflecting Wnt activity in a hind limb associated tumor. The same tumor is shown 3, 4 and 7 weeks after *apc* TALEN injection. Active Wnt signalling is detected in the entire tumor in the initial stages (A) but gets restricted to undulated strands at the edge of the tumor in the later stages (B, C). (D) Brightfield and (E) fluorescent image of epidermoid cysts (red arrows) showing active Wnt signalling. Fluorescence of the neighboring skin is due to autofluorescence. (F) Brightfield and (G) fluorescent images of an exposed desmoid tumor (red arrowhead) with the skin partially removed showing active Wnt signaling in comparison to the neighboring exposed muscle (white arrows). The white arrowhead indicates autofluorescent skin overlying the tumor.

**Figure 6:**
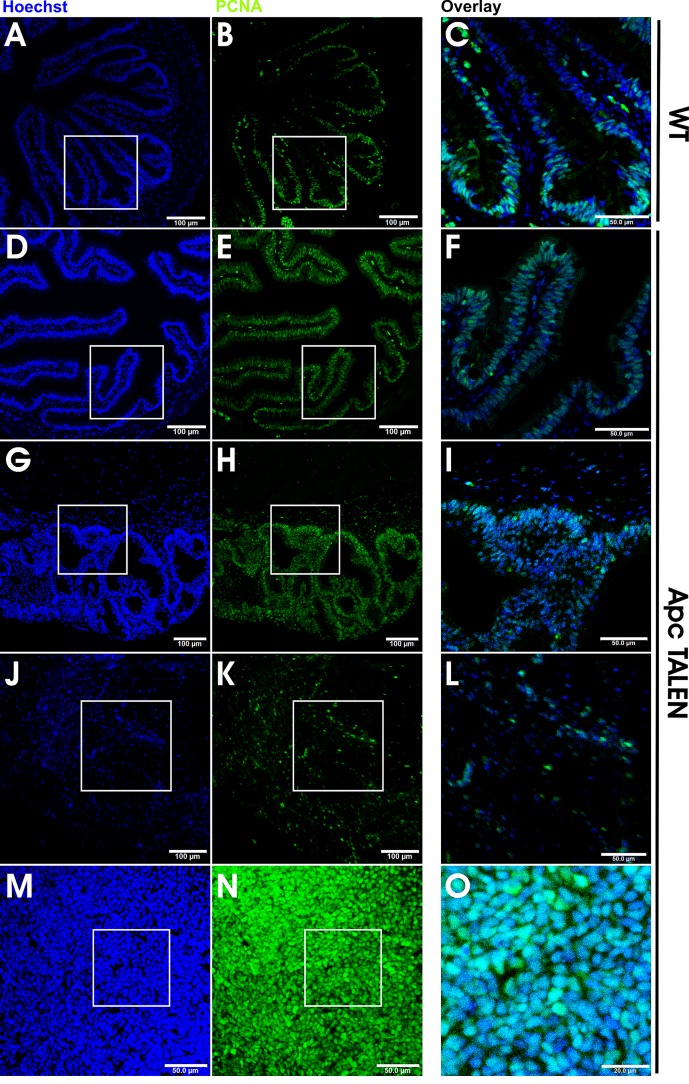
Increased and mislocalized proliferation in *apc* TALEN induced tumors Hoechst (left panels) and PCNA (middle panels) double staining; magnified overlays in right panels. (A, B, C) In WT small intestine PCNA staining is primarily localized at the base of the folds and largely absent in the crests. (D, E, F) Small intestine of *apc* TALEN injected froglet showing the presence of PCNA staining along the entire trough-crest axis. (G, H, I) Epidermoid cysts showing a high number of proliferating PCNA positive cells. (J, K, L) Desmoid tumor displaying a large fraction of PCNA positive nuclei. (M, N, O) Hind limb-associated tumor with almost all nuclei staining positive for PCNA indicating a very high proliferation rate.

### Medulloblastomas

Some tadpoles injected with *apc* TALENs showed abnormal swimming behavior, including defects in balance. Abnormalities in the brain were apparent externally (Fig. 2F) or upon dissection. Histological examination of these tadpoles showed neoplasms that closely resemble medulloblastoma (Fig. 4G,H), which is another tumor type observed in a subset of FAP patients [22].

### Retinal hyperproliferation

A fraction of tadpoles injected with the *apc* TALENs showed enlargement of the eyes. Histology showed abnormal organization of the retinal cell layers and the lens. The localized expansion of the cells of the neural retina adopted rosette-like structures and ectopically localized retinal pigment epithelium (Fig. 3C,D). These retinal malformations may be linked to Congenital Hypertrophy of the Retinal Pigment Epithelium (CHRPE), which is frequently observed in FAP patients [22].

### Wnt/β-catenin signaling is active in *apc* mutant tumors

To investigate whether the mosaic mutations in the MCR of *apc* resemble also functionally the situation in mammals and induce neoplasia with a hyperactive Wnt/β-catenin signaling pathway, we injected the *apc* TALENs in embryos obtained from a transgenic *X. tropicalis* Wnt reporter line that we previously generated and characterized [23, 24]. GFP signals were analyzed in living tadpoles and in freshly dissected desmoid tumors. The most striking increase in GFP signal was observed in the fast-growing hind limb tumors (Fig. 5A-C) and in the epidermoid cysts (Fig. 5D,E). Remarkably, in the limb tumors the signal was especially strong in the peripheral region and was organized in undulated continuous strands (Fig. 5C). Histological examination and staining for phosphorylated histone 3 showed that the areas of highest GFP signal also had the highest cell density. It is unclear whether the intense GFP signal was due to a stronger Wnt response or primarily reflected the density of the cells in the tumor. Also the desmoid tumors showed increased GFP signals, primarily visible when the skin, which shows autofluorescence, was removed and the tumor was exposed (Fig. 5F,G). The fluorescent signal was weaker compared to the epidermoid cysts and the limb tumors but this is probably due to the fact that the fibromatous desmoid tumors are very rich in extracellular matrix but particularly low in cell density. Together, these data show that Wnt/β-catenin signaling is increased, at least in the three types of tumors that were analyzed.

**Figure 7:**
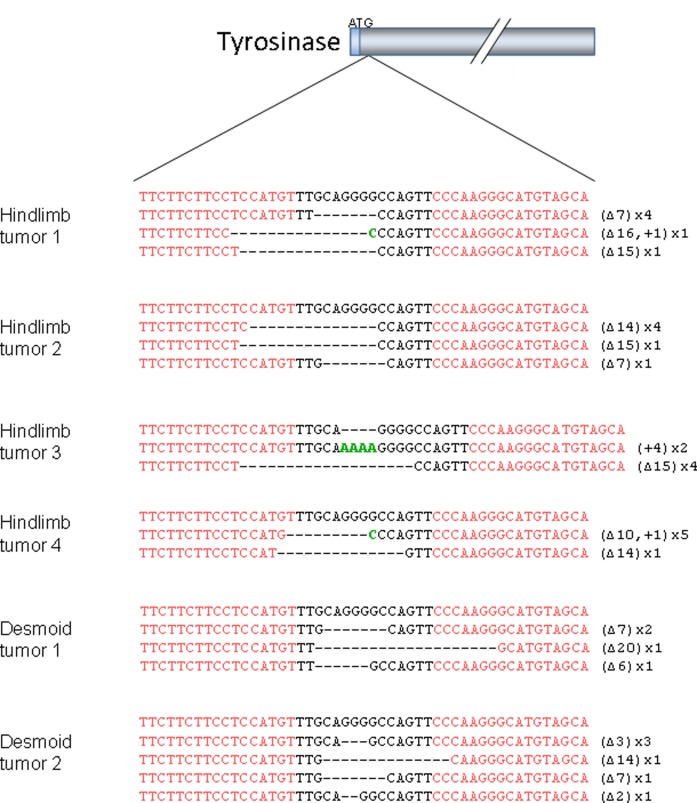
Combined bi-allelic targeting of the *tyr* and *apc* genes in *X. tropicalis* tumors INDELs found in the *tyr* gene in *apc* mutant tumors developing after co-injection of *apc* (40 pg) and *tyr* TALEN (300 pg) mRNAs. Note that in contrast to the INDELs observed in the *apc* locus (see Fig. 1C), deletions in the *tyr* locus can be in frame, *e.g.* Δ3 or Δ15.

### *apc* mutant tumors show abnormal proliferation profiles

To further examine whether TALEN-driven *apc* mutation affects cell proliferation, we performed proliferating cell nuclear antigen (PCNA) analysis on sections from intestines, epidermoid cysts, and the desmoid and hind limb tumors. As described previously [25], cell proliferation in the froglet intestine is restricted to the lower third of the trough-crest axis, which resembles the crypt-villus organization in mammals (Fig. 6A-C). In the intestines of frogs injected with *apc* TALEN, PCNA staining was uniform both in troughs and in crests of the longitudinal folds (Fig. 6D-F). This is reminiscent of the situation in mice after loss of *Apc* and in humans carrying familial *APC* mutations [26,28].

Increased PCNA staining was also apparent in other tumor types that we analyzed by immunostaining, namely the epidermoid cysts (Fig. 6G-I) and desmoid tumors (Fig. 6J-L). The limb tumor in particular showed rapid cell proliferation, with > 90% of the cells showing increased PCNA staining (Fig. 6M-O).

### Multiplexed gene disruption in *Xenopus* tumors

A major asset of our model is the possible identification and characterization of genes that cooperate with APC-driven tumor formation or progression. To investigate whether we can obtain double bi-allelic gene disruptions in the tumors, the TALEN pair targeting the *apc* MCR was co-injected in two-cell stage embryos with a TALEN pair targeting the non-selective Tyrosinase (*tyr*) gene [29]. Tadpoles were grown until they developed limb and desmoid tumors. Tumor samples were obtained and DNA was extracted. The targeted region of the *tyr* gene was amplified by PCR and subcloned into pTOPO plasmids. All tumor samples contained only *tyr* alleles with INDEL mutations (Fig. 7), in addition to the obligate mutant *apc* alleles (data not shown). These results show that multiplexed bi-allelic gene targeting is straightforward in the developing *Xenopus* tumors.

## DISCUSSION

We describe here a new and representative model for both familial and sporadic cancers associated with mutations in the *APC* tumor suppressor gene. Our *Xenopus* model develops at least three tumor types that are also found in human FAP patients: intestinal neoplasias, desmoid tumors, and medulloblastomas. Moreover, histological distortions in the *Xenopus* retina bear a resemblance to FAP-associated CHRPE. Two additional tumor types could not be linked to FAP, *i.e.* the limb tumors and the PNET-like outgrowths in the epidermis. These tumors might be development-related neoplasia, which are due to the early bi-allelic *apc* mutations that are inherent to the TALEN approach, and which do not occur in human FAP patients.

The vast majority of the *Xenopus* tumors contained only mutant *apc* alleles. For the epidermoid cysts, the desmoid tumors and the hind limb tumors we demonstrated increased activation of the Wnt/β-catenin signaling pathway. Increased cell proliferation, as revealed by PCNA staining, was evident in all the tumor types. Interestingly, while the intestine was neoplastic, with proliferation extending throughout the crypt−villus axis, we did not observe genuine adenomas. We believe that this was due to the distinct histological organization of the post-metamorphic intestine of *Xenopus*, where cells are organized in continuous extended folds. This means that a clone of hyperproliferating cells would not be trapped locally, as would be the case in mammalian crypts. Instead, they can move sideways and cause expansion of the folds. This also explains the abnormal curly appearance of these folds (see Fig. 2B).

Cancer associated with *APC* mutations has already been extensively modeled in mice, both in spontaneous models such as the Apc^Min^ mouse and in models using inducible tissue-specific *Apc* truncations [3]. A zebrafish model using ENU-induced mutation of the *Apc* MCR region was also described [30]. Heterozygous mutant fish effectively developed adenomas associated with increased Wnt/β-catenin signaling [4]. However, this only happened after 15 months in only a few of the adult animals [4]. Therefore, a faster aquatic model for APC-associated cancer with a higher penetrance, such as the *Xenopus* model described here, will be a major asset to the field.

Indeed, a major benefit of our model is the high penetrance and reproducibility of tumor formation and the rapid induction of the neoplasms, with the first desmoid tumors visible in six weeks. This opens the door for a myriad of applications including genotype-phenotype analysis by designing TALENs to different regions of the *apc* gene [31-33]. In addition, the rapid induction and reproducibility of tumor formation, combined with the aquatic habitat of the tadpoles and froglets, might permit fast and efficient pre-clinical screening of candidate compounds. It will be interesting to validate our model with experimental compounds or existing, clinically approved drugs, that were recently shown to interfere with APC associated intestinal or desmoid tumor formation [34-36].

In addition, we believe that our new *Xenopus* tumor model may be especially useful for identifying or characterizing possible effectors or modifiers of APC mediated tumor formation [37-39]. Also, analysis of tumor suppressors that cooperate with APC, or genes involved in tumor progression, would be a valuable extension of our model. The possibility of generating tumors with bi- allelic mutations of two independent genes –as shown here for the *apc* and the *tyr* genes– is an important benefit of our model. This approach is especially interesting for *X. tropicalis*, which is a tetrapod and a true diploid. In comparison, such approach would be less feasible in zebrafish, where about 30% of the genes are duplicated due to a genome duplication event that occurred during the phylogenetic development of the ray-finned fishes [40]. In addition, the very high synteny between the human and the *X. tropicalis* genomes facilitates the identification of the frog orthologs for most human genes of interest [14]. Evidently, *Xenopus* does not have the benefit of numerous existing mutant lines generated in zebrafish by the TILLING project and the Zebrafish Mutation project [10, 11]. However, considering current methods for efficient and cheap genome editing by using TALENs and CRISPR/Cas9, this shortcoming is expected to be easily overcome.

In summary, we generated the first genuine genetic tumor model in *X. tropicalis* using TALEN-mediated targeting of the tumor suppressor gene *apc*. Our approach would probably be feasible for other cancer-related genes, especially tumor suppressors. This should be an incentive for further use of this tetrapod organism for modeling human genetic diseases and cancer and as a model for pre-clinical drug screening. *Xenopus* models can become a powerful supplement to the currently frequently used murine and zebrafish models.

## MATERIALS AND METHODS

### TALEN design, cloning and mRNA transcription

A TALEN pair targeting the MCR of *Xenopus tropicalis apc* was designed using the TAL Effector Nucleotide Targeter 2.0 software [18], with spacer length and repeat array lengths all at 16 bp. The selected TALEN pair (Fig 1A) was cloned using the Golden Gate Cloning protocol [16], but using the pCS2-Flag-TALEN-ELD/KKR vectors as the final backbones for the TALEN pair [41]. The TALEN pair targeting the *tyr* gene in *X. tropicalis* was a kind gift from Dr. Enrique Amaya [29]. These vectors were linearized using *Not*I, and mRNA was transcribed using the mMessage machine sp6 kit (Ambion). Finally, capped mRNA was extracted with phenol/chloroform, precipitated with isopropanol, and dissolved in nuclease-free water.

### *Xenopus tropicalis* microinjection

Wild type and Wnt-reporter transgenic male and female *X. tropicalis* were primed 2 days before injection with 10 and 20 IU of human chorionic gonadotropin (hCG), respectively. Natural matings were set up in the morning, after boosting males and females with 100 and 150 IU of hCG, respectively. Embryos were collected and the jelly coat was removed using a 2% cysteine solution in 0.1X MMR (Marc's Modified Ringers solution). Embryos were injected unilaterally at the 2-cell stage with 40 or 300 pg/embryo of *apc* TALEN mRNA. At the 8-cell stage, single blastomeres (animal-ventral, animal-dorsal, vegetal-ventral or vegetal-dorsal) were injected with 20 pg of *apc* TALEN mRNA. For the multiplexing experiment, *tyr* TALEN RNA and *apc* TALEN RNA were coinjected at a concentration of 300 and 40 pg/embryo, respectively.

All experiments were performed in accordance with relevant guidelines and regulations of Ghent University, Faculty of Sciences.

### DNA extraction and sequencing

Tadpoles and dissected tumors were incubated overnight at 55°C in lysis buffer (50 mM Tris pH 8.8, 1 mM EDTA, 0.5% Tween-20, 200 μg/ml proteinase K). Genomic regions encompassing the *apc* and *tyr* TALEN cut sites were PCR amplified using specific primer pairs (*apc*: 5′-CATCCTAACTCTGCCCAA-3′, 5′-ATAATGTTCTGGTGGGCT-3′; *tyr*: 5′-ACATATCAATCACCCCAACTC-3′, 5′-TCTATCGTCAACCCCAGTG-3′) and cloned using the pTOPO Zero Blunt® kit (Invitrogen). After transformation, plasmids were extracted from individual bacterial colonies and sequenced by the Sanger method.

### Imaging, histology and immunohistochemistry

Living and dissected tadpoles and froglets were imaged on a Carl Zeiss StereoLUMAR.V12 stereomicroscope. Tissues and tumors were fixed in 4% PFA (paraformaldehyde), dehydrated, and embedded in paraffin. Sections of 5 μm were rehydrated and stained with hematoxylin and eosin for histological examination. For PCNA immunohistochemistry, 5-μm sections were pressure cooked in citrate buffer (10 mM citric acid, 0.05% tween-20, pH 6) for antigen retrieval and blocked in 3% goat serum, 1% BSA, 0.1% tween-20 in PBS. Slides were incubated overnight with PCNA antibody (Clone PC10-Dako) at 4°C. Secondary goat anti-mouse dylight-488 conjugated antibody was used for detection. Fluorescent tumor sections were imaged on a Leica TCS LSI zoom confocal microscope.
